# Intact priors for gaze direction in adults with high-functioning autism spectrum conditions

**DOI:** 10.1186/s13229-016-0085-9

**Published:** 2016-04-15

**Authors:** Philip J. Pell, Isabelle Mareschal, Andrew J. Calder, Elisabeth A. H. von dem Hagen, Colin W.G. Clifford, Simon Baron-Cohen, Michael P. Ewbank

**Affiliations:** Medical Research Council, Cognition and Brain Sciences Unit, Cambridge, UK; School of Biological and Chemical Sciences, Psychology Department, Queen Mary University of London, London, UK; Autism Research Centre, Department of Psychiatry, University of Cambridge, Cambridge, UK; School of Psychology, Cardiff University, Cardiff, UK; School of Psychology, UNSW Australia, Sydney, NSW Australia; MRC Cognition and Brain Sciences Unit, 15 Chaucer Road, Cambridge, CB2 7EF UK

**Keywords:** Autism, Bayesian priors, Gaze perception, Autistic traits, Uncertainty

## Abstract

**Background:**

Autism Spectrum Conditions (ASC) are associated with a range of perceptual atypicalities, including abnormalities in gaze processing. Pellicano and Burr (Trends Cogn Sci 16(10):504-10, 2012) have argued that these atypicalities might be explained within a Bayesian framework, in which perception represents the combination of sensory information with prior knowledge. They propose that the Bayesian priors of individuals with ASC might be attenuated, such that their perception is less reliant on prior knowledge than neurotypical individuals. An important tenet of Bayesian decision theory is that increased uncertainty about incoming sensory information will lead to a greater influence of the prior on perception. Consistent with this, Mareschal et al. (Curr Biol 23(8):717-21, 2013) showed that when noise is added to the eyes of a face (increasing uncertainty about gaze direction), gaze is more likely to be perceived as direct.

**Methods:**

We adopted the same paradigm as Mareschal et al. to determine whether the influence of a prior on gaze perception is reduced in neurotypical participants with high numbers of autistic traits (experiment 1) and in individuals with a clinical diagnosis of ASC (experiment 2). Participants were presented with synthetic faces and asked to make a judgement about the relative gaze directions of the faces. Uncertainty about gaze direction was manipulated by adding noise to the eyes of a face.

**Results:**

Consistent with previous work, in both experiment 1 and experiment 2, participants showed a bias towards perceiving gaze as direct under conditions of uncertainty. However, there was no evidence that the magnitude of this bias was reduced either in the ASC group or in neurotypical controls with a high number of autistic traits.

**Conclusions:**

Our findings challenge the attenuated priors theory of perception in ASC (Trends Cogn Sci 16(10):504-10, 2012) and related proposals (Trends Cogn Sci 17(1):1, 2013, Front Hum Neurosci 8:302, 2014), and suggest priors for gaze direction are intact in high-functioning ASC.

## Background

Autism Spectrum Conditions (ASC) are characterised by impairments in social communication and social interaction, alongside restricted, repetitive patterns of behaviour and unusually narrow interests, and difficulty adjusting to unexpected change [1]. Research indicates that individuals with ASC often show sensory and perceptual atypicalities, including both hyper- and hypo-sensory sensitivity [2], reduced perception of coherent motion, superior processing of embedded figures and, in some instances, reduced susceptibility to visual illusions [3]. Atypicalities in the perception of social stimuli include difficulties in the processing of facial identity [4] and eye gaze [5], although recent work has found evidence of typical response patterns in ASC in other areas of social perception [6, 7].

Pellicano and Burr [8] have argued that perceptual atypicalities found in ASC might be explained within a Bayesian decision framework. According to the Bayesian decision models of perception, our perceptual system generates the most probable interpretation of the environment by combining sensory information with prior expectations. Pellicano and Burr propose that the priors of individuals with ASC might be attenuated (‘hypo-priors’) such that previous experience will have less influence on perception. In this sense, they suggest that individuals with ASC can be considered as perceiving the world more ‘accurately’ than neurotypical controls (although see [9] for an alternative interpretation wherein ‘hypo-priors’ could lead to less accurate perception) and that attenuated priors are consistent with evidence of superior visual perception in ASC [10–12]. In a similar approach, Lawson et al. [13] explain perceptual atypicalities in ASC within the neural instantiation of Bayesian inference—predictive coding [14]—  (see also Friston et al., [15]), and suggest that perceptual atypicalities in ASC can be explained as aberrant encoding of precision, i.e. an imbalance between sensory evidence and prior beliefs, with greater weight being applied to sensory evidence.

If priors are atypical in ASC, this should manifest in differences in perceptual phenomena that emerge as a consequence of previous visual experience. Perceptual adaptation is a clear form of experience-dependent plasticity, which, within a Bayesian framework, is driven by a mismatch between sensory experience and prior expectations leading to a change in perception [16]. Consistent with the attenuated priors theory of perception in ASC, perceptual aftereffects for both facial identity and eye gaze have been shown to be reduced in children and adolescents with ASC [17, 18]. However, more recent work found no evidence of reduced facial aftereffects in adults with ASC [19–21]. At a neural level, repetition of a stimulus leads to habituation (a reduction in neural activity), and a study by Kleinhans et al. [22] found that individuals with ASC showed attenuated habituation of the amygdala to repeated presentations of faces.

Visual illusions have also been cited as an example of how priors are used to formulate the most likely interpretation of noisy or ambiguous sensory input [23]). In accordance with the attenuated priors theory, individuals with ASC show reduced susceptibility to visual illusions (e.g. Happé [24]), although the reliability of this effect has been challenged [3]. An important tenet of Bayesian decision theory is that increased uncertainty about incoming sensory information will lead to an increased influence of prior expectations on perception. Mareschal et al. [25] showed that when noise is added to the eyes of a face (thus increasing uncertainty about gaze direction), gaze is more likely to be perceived as direct (and see also [26]). This suggests that humans have a prior expectation that eye gaze is directed towards them.

The aim of this study was to test the attenuated priors theory of perception in ASC in a visual social perception task by determining: (1) whether the influence of a prior on gaze perception is reduced in individuals with a high number of autistic traits, relative to those with a low number of autistic traits, within a neurotypical sample (experiment 1) and (2) whether individuals with a formal diagnosis of ASC show evidence of reduced influence of gaze priors relative to age and IQ-matched controls (experiment 2). One proposal is that ASC is an extreme end of a normal distribution [27, 28] that extends into the neurotypical population (and see also Ruzich et al. [29] for a systematic review of autistic traits in the non-clinical population). Moreover, individual differences in autistic traits have been shown to predict performance in neurotypical and clinical populations on a number of tasks that are impaired in ASC, including reading others’ mental states [30], face recognition [31], and gaze processing [32]. Recent work has also shown that repetition suppression to faces and non-faces in occipitotemporal cortex is diminished as a function of increasing autistic traits in a neurotypical population [33]. Thus, investigating the effect of autistic traits in neurotypical participants provides a complementary approach to the study of individuals with a clinical diagnosis of ASC.

Following Mareschal et al. [25], we predicted that the neurotypical groups would show evidence of a prior for direct gaze. However, if this effect is diminished in participants with greater numbers of autistic traits, and also significantly weaker in the ASC group relative to controls, this would provide support for the attenuated priors theory of perception in ASC. In a modification to the original paradigm, we calibrated the noise contrast for each participant based on their performance on a baseline gaze discrimination task. This enabled us to equate the effectiveness of the noise across participants; thus, if gaze priors are similar for the control and ASC group, then the absence of a difference in gaze priors is unlikely to be explained by differences in sensory noise. To our knowledge, this study represents the first direct test of the theory of attenuated priors in ASC in high-level social perception.

## Experiment 1

### Methods

#### Participants

Thirty-four participants (18 female) completed the experiment. All were right-handed and had normal or corrected to normal vision. Participants completed the Autism Spectrum Quotient (AQ), a 50-item validated measure of autistic traits that is suitable for use in neurotypical and clinical populations [27], and the Social Responsiveness Scale (SRS) (SRS-second edition), a quantitative measure for identifying ASC symptoms in neurotypical and clinical adult populations [34]. Participants were assigned to either a low or high AQ group using a median split of total AQ score (median = 13.5) (see Table 1 for participant information for each group). Previous work found that 79 % of individuals with high-functioning autism/Asperger syndrome scored above 32 on the AQ [27]; however, the AQ is not a diagnostic measure [27] and no participants had a clinical diagnosis of an ASC. Similarly, a total raw score above 67 on the SRS is associated with ‘deficiencies in reciprocal social interaction’, but SRS scores alone are not diagnostic of an ASC. The study was approved by the Cambridge Psychology Research Ethics Committee (CPREC.2013.57). All volunteers were naive to the aims and objectives of the experiment, provided written informed consent and were paid for participating.

**Table 1:** Demographics for participants in experiment 1 and experiment 2

	Experiment 1			Experiment 2		
	Low AQ	High AQ	Comparison	Control	ASC	Comparison
N	17	17		11	11	
Sex (n male to n female)	8:9	8:9		9:2	9:2	
Age (years)						
Mean (SD)	25.19 (4.72)	29.65 (8.82)	*p* = .07	32.27 (7.17)	32 (9.23)	*p* = .94
Range	19–36	21–40		22–44	18–45	
AQ						
Mean (SD)	7.75 (2.57)	23.24 (4.8)	*p* < .001	12.17 (4.73)	40.73 (5.68)	*p* < .001
Range	2–11	16–30		4–19	30–48	
SRS						
Mean (SD)	25.5 (12.37)	57.35 (21.8)	*p* < .001	36.2 (25.59)	109 (23.43)	*p* < .001
Range	8–50	21–105		11–81	60–133	
Full-scale IQ						
Mean (SD)	N/A	N/A		127.18 (10)	126.45 (13.62)	*p* = .89
Range	N/A	N/A		113–141	99–143	

SRS scores represent raw total score

*N/A* not available

#### Stimuli

Stimulus generation, stimulus presentation and response recording were controlled by MATLAB (Mathworks Ltd) and PsychToolbox run on a Dell Optiplex 745 computer. Stimuli were viewed at a distance of 57 cm and displayed on a calibrated Dell P791 CRT monitor (1024 × 768, 75 Hz) driven by the computer’s built-in ATI Radeon X1300 Pro graphics card. Grayscale synthetic neutral faces, subtending, on average, 15.1° × 11.2° visual angle, were created using Daz software (http://www.daz3d.com/). The deviation of each eye was independently controlled with MATLAB procedures, giving precision down to the nearest pixel for horizontal eye rotations. Eight grayscale faces (four female) were used in the mixed gaze discrimination task. Fractal noise (1/f amplitude spectrum) was added to the eyes in noise conditions, and the contrast between pupil and sclera varied according to performance on the baseline gaze discrimination task (see below).

### Procedure

#### Baseline noise threshold

To equate performance across participants, noise was added to the eyes of computer-generated faces and participants were asked to judge whether the gaze in a forward facing head (15°) was to their left or to their right. A Psi Bayesian adaptive procedure was employed which estimates the choice of the next trial stimulus level based on the responses to all the previous trials. The procedure optimises the stimulus placement, while being more robust to changes in slope, and therefore is well-suited to test clinical populations. It converges at the end of 30 trials on the pupil/sclera contrast level leading to 80.3 % correct discrimination [35]. Participants were given instructions on the task and then completed four runs of 30 trials back to back. The median noise threshold from the four runs was used in the mixed gaze discrimination task.

#### Mixed gaze discrimination

**Fig. 1:**
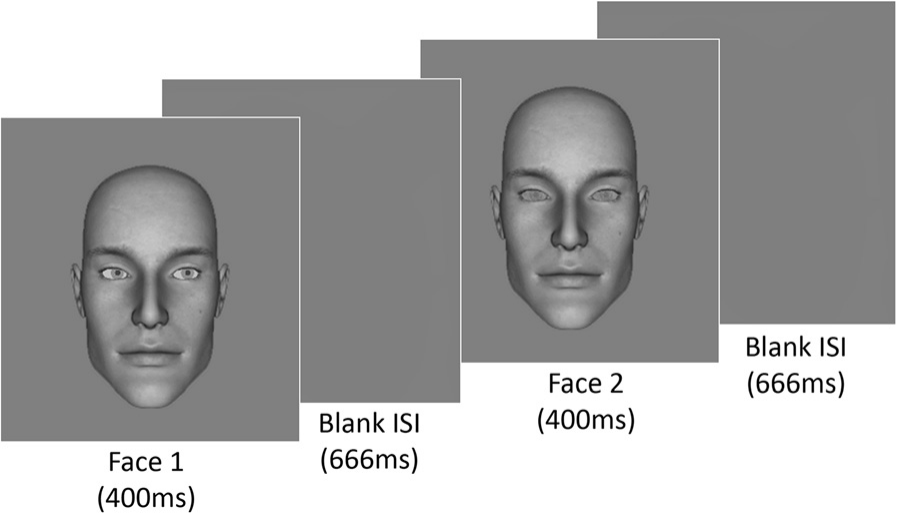
Experimental procedure showing a sample trial (with noise added to the second face) used in experiments 1 and 2. The same identity face was presented twice, and participants were required to indicate the direction of gaze in the second interval relative to the first

Observers discriminated the direction of gaze between two same identity faces in a two-interval forced-choice task (Fig. 1). One was the ‘test’ face with a fixed gaze deviation (9° left or 9° right) and the other was a ‘comparator’ face with an offset (−20°, −10°, −5°, −2°, 2°, 5°, 10°, 20°) added to the fixed test deviation, and the observer’s task was to indicate whether the direction of gaze of the face in the second interval was averted to the left or to the right compared to the direction of gaze of the face in the first interval. Noise was added to the eyes of one of the two faces at the noise contrast determined in the baseline noise threshold task. The noisy face occurred with equal frequency in the two intervals, and noise was added either to the ‘test’ or ‘comparator’ with equal occurrence. Each offset level was sampled 12 times in a run, and a logistic function was fit to the data to obtain an estimate of the gaze offset producing 50 % right responses (‘bias’). After a short practice (15 trials from the first run), two runs were performed back-to-back with a short break in between. The order of test deviations (−9 vs. 9) was counterbalanced within AQ groups.

### Results

Following Mareschal et al. [25], linear fits were made through bias scores at the two test deviations (−9° and 9°). The direct gaze bias identified by Mareschal et al. [25] is characterised by a significant difference in the slopes of the fits, depending on whether noise was added to the test or to the comparator, with more positive slopes for the comparator noise condition and more negative slopes for the test noise condition. To test for this effect and potential effects of autistic traits, slopes were entered into a 2 × 2 ANCOVA with noise condition (test, comparator) as a within-participants factor and AQ group (high AQ, Low AQ) as a between-participants factor. Threshold (individual thresholds from the baseline noise threshold task) was included as a covariate to examine whether the relationship between slope and threshold differs across noise conditions.

**Fig. 2:**
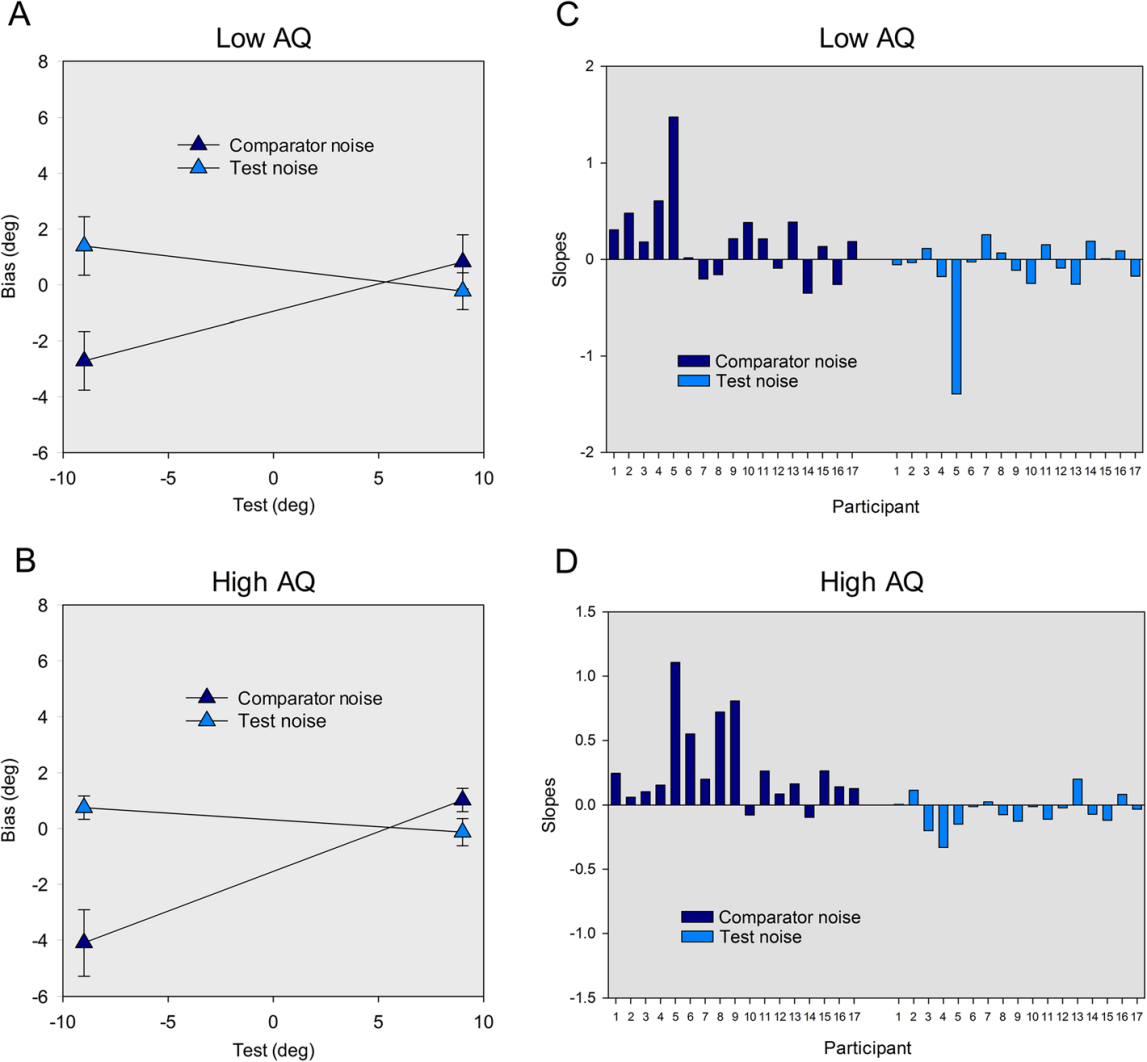
Experiment 1. Average bias scores for (**a**) the low AQ group and (**b**) the high AQ group when the test contained noise (*filled diamonds*) and when the comparator contained noise (*crossed diamonds*) at each test deviations −9° and 9°. Individual slopes of the biases for all participants in the (**c**) low AQ group and (**d**) the high AQ group when the test contained noise (*filled bars*) and when the comparator contained noise (*crossed bars*)

The ANCOVA revealed a significant main effect of noise condition (*F*(1, 31) = 11.27, *p* = .002, *η*_*ρ*_^2^ = .27), but no effect of AQ group (*F*(1, 31) = 2.23, *p* = .15) and, crucially, no significant interaction between noise condition and AQ group (*F*(1, 31) < 1, *p* = .89) (Fig. 2a, b). These results indicate that slopes in the comparator noise condition are significantly more positive than in the test noise condition, consistent with a bias towards perceiving uncertain gaze directions as direct (see also Fig. 2c, d for a summary of individual participants’ slopes). However, there was no evidence that the magnitude of this effect is modulated by autistic traits. The ANCOVA also revealed an effect of threshold (*F*(1, 31) = 4.58, *p* = .04, *η*_*ρ*_^2^ = .13) and a significant interaction between threshold and noise condition (*F*(1, 30) = 7.44, *p* = .01, *η*_*ρ*_^2^ = .14). To further explore this finding, separate Person’s correlations were used to examine the relationship between threshold and slope in each noise condition. There was a significant negative relationship between slope and threshold in the comparator noise condition *r* = −.47, *p* = .005 (as threshold decreases, comparator noise slope becomes more positive), and a trend towards a positive relationship in the test noise condition *r* = .31, *p* = .07 (as threshold decreases, test noise slopes becomes more negative). These results indicate that the strength of the direct gaze bias (as signified by increasingly positive comparator noise slopes or increasingly negative test noise slopes) increases as stimulus noise increases, consistent with a Bayesian decision framework. Importantly, the average gaze discrimination thresholds did not differ between the AQ groups (low AQ = .202, high AQ = .201) *t*(32) < 1, *p* = .99) indicating that effective noise is well-matched across groups.

As the mean age of the high AQ group was marginally greater than the low AQ group, we performed an additional ANCOVA including age as a covariate of no interest. Again, this revealed a significant main effect of noise condition (*F*(1, 30) = 4.26, *p* = .049, *η*_*ρ*_^2^ = .13), no effect of AQ group (*F*(1, 30) = 1.35, *p* = .26) and no significant interaction between noise condition and AQ group (*F*(1, 30) < 1, *p* = .98).

Inspection of Fig. 2c reveals that one participant (P5) in the low AQ group showed a particularly large bias. To investigate whether the results for this individual have a disproportionate effect on the overall results of the ANCOVA, this test was repeated without P5. All significant effects found in the previous analysis remained significant (*p* < .05), and again, we found no significant interaction between noise condition and AQ group (*F*(1, 30) = 2.35, *p* = .14). Finally, Pearson’s correlation analysis revealed no evidence of a significant relationship between total SRS scores and slopes for each noise condition, whether or not P5 was included in the analysis (*r* < .17, *p* > .36).

#### Bayesian analysis

Statistical tests using Bayes factors (BF) were conducted to quantify the strength of the evidence for the null hypothesis (no difference between groups) versus the alternative (low AQ > high AQ). It is important to conduct Bayesian analysis as studies claiming no difference between groups typically rely on null hypothesis significance testing to imply the null hypothesis is true, without quantifying the degree to which the evidence supports the null hypothesis. As a point of reference: BF_10_ of 1–3 indicates weak/anecdotal evidence for the alternative hypothesis; BF_10_ of 3–10 corresponds to positive/substantial support for the alternative hypothesis and BF_10_ >10 indicates positive/strong evidence for the alternative hypothesis [36, 37]. Using JASP (https://jasp-stats.org) [38], a Bayesian independent *t* tests was conducted, with default prior scales for each experiment, comparing difference scores (noise > comparator) between groups. The results revealed ‘substantial’ evidence in favour of the null hypothesis (BF_10_ = 3.3).

### Discussion

Previous research suggests that humans have a prior expectation that gaze is directed towards them [25]. The purpose of this experiment was to test whether the strength of this effect is influenced by individual variation in autistic traits in a neurotypical sample. Participants completed a short baseline noise threshold task to determine the contrast level (in the context of fractal noise) required to discriminate the left from the right gaze at 80.3 % correct performance levels. In a subsequent mixed discrimination task, they had to compare the gaze direction of a face with noise added to the eye region (tailored to their contrast threshold) with that of a face with no noise added to the eyes. Consistent with the results of Mareschal et al. [25], we found that psychometric response functions were shifted in opposite directions depending on whether the noise was added to the test face (with a fixed gaze direction) or the comparator face (with a varying gaze offset). This bias towards perceiving direct gaze was found for individuals with high or low numbers of autistic traits. Furthermore, there was no evidence of a difference in the magnitude of the direct gaze bias between groups. Importantly, we found no difference in the overall threshold across groups indicating that these results cannot be explained in terms of differences in sensitivity to noise. Moreover, direct gaze bias was negatively correlated with threshold, which supports a Bayesian framework wherein higher noise (i.e. higher uncertainty) is associated with increased influence of the prior.

## Experiment 2

### Background

In experiment 1, we found no evidence that the direct gaze prior is related to increasing autistic traits in a neurotypical population, a result that appears inconsistent with the attenuated priors theory of perception in ASC. However, it is possible that attenuated priors are only a characteristic of individuals with clinical diagnosis of an ASC or that any attenuation of priors in neurotypical individuals is too small or inconsistent to be detected with our paradigm. To address this potential limitation, in experiment 2, we used the same paradigm with a group of individuals with a diagnosis of high-functioning autism (ASC) and a group of neurotypical controls matched on IQ, age and sex. If ASC is associated with attenuated priors, then the ASC group should show a smaller bias towards perceiving gaze as direct relative to controls.

### Methods

#### Participants

For the neurotypical control group, 11 participants (2 females, mean age 32.27) were recruited from the MRC CBU volunteer panel. All were right-handed and had an AQ <20 (see Table 1). The ASC group consisted of 11 volunteers (2 females, mean age 32) recruited via the Cambridge Autism Research Centre. All had written confirmation of an independent diagnosis of an ASC (high-functioning autism or Asperger syndrome) by a qualified clinician using DSM-IV criteria [39]. In addition, six had a confirmed diagnosis using the Adult Asperger Assessment (AAA) [40] and two using the Autism Diagnostic Observation Schedule (ADOS) [41]. Individuals with any other current psychiatric diagnosis (other than ASC in the ASC group) were excluded from the study. All participants completed the Wechsler Abbreviated Scale of Intelligence – second edition (WASI-II) [42] and all scored >99. All participants were right-handed, had normal or corrected to normal vision and were naive to the aims and objectives of the experiment. No participants were taking psychotropic medication at the time of the study. All participants also completed the AQ and the SRS, and as expected, the ASC and control groups differed significantly on both measures (see Table 1). The study was approved by the Cambridge Psychology Research Ethics Committee (CPREC.2013.57). All volunteers provided written informed consent and were paid for participating.

#### Procedure

The procedure was identical to that of experiment 1. The order of test deviations (−9 vs. 9) was counterbalanced within groups.

### Results

**Fig. 3:**
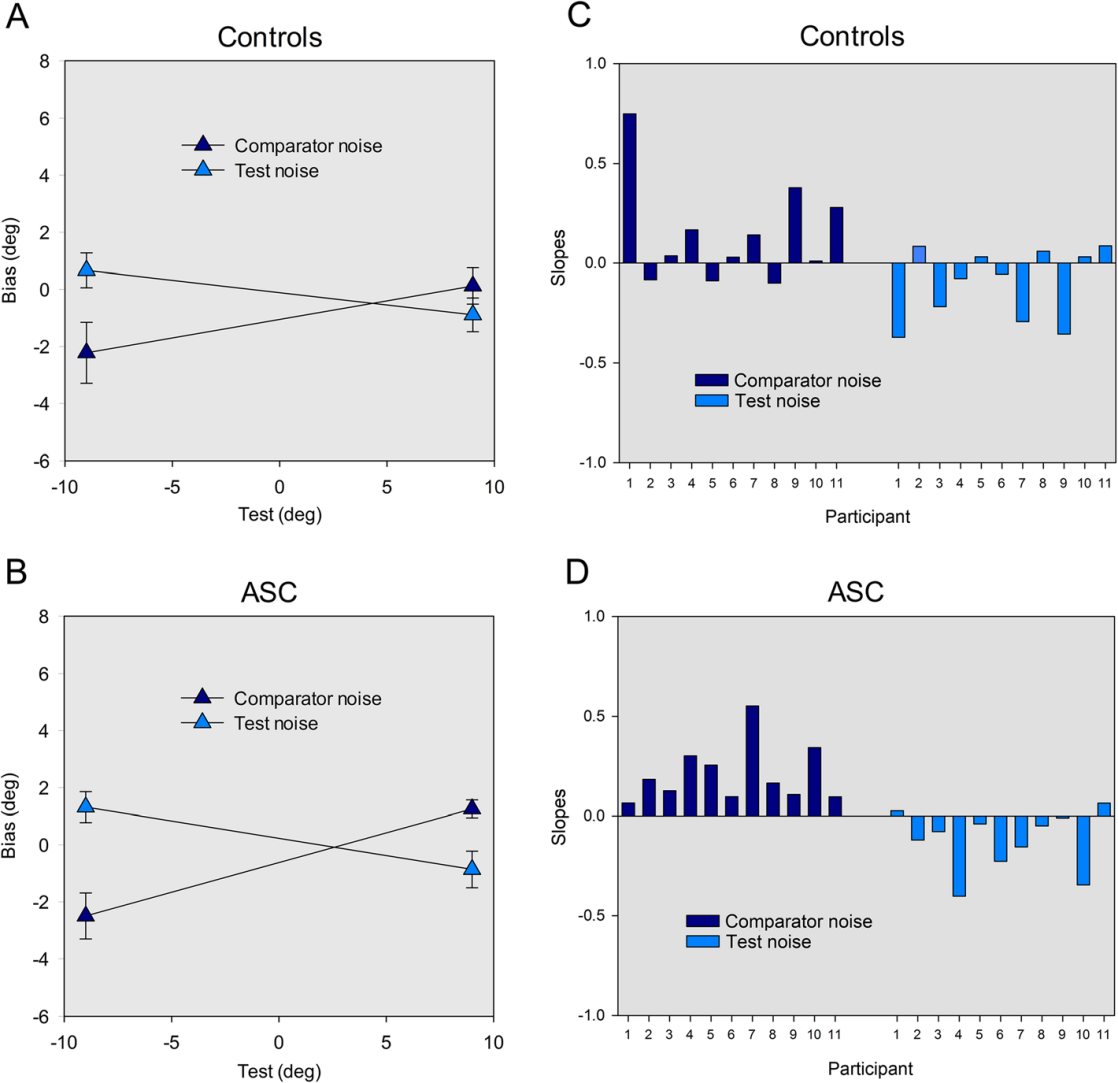
Experiment 2. Average bias scores data for (**a**) the control group and (**b**) the ASC group when the test contained noise (*filled diamonds*) and when the comparator contained noise (*crossed diamonds*) at each test deviations −9° and 9°. Individual slopes of the biases for all participants in the (**c**) control group and (**d**) the ASC group when the test contained noise (*filled bars*) and when the comparator contained noise (*crossed bars*)

As in experiment 1, to test for a direct gaze bias and group differences, slopes were entered into a 2 × 2 ANCOVA with noise condition (test, comparator) as a within-participants factor and group (ASC, control) as a between-participants factor. Threshold (baseline noise threshold) was included as a covariate. The results revealed a main effect of noise condition (*F*(1, 19) = 20.26, *p* < .001, *η*_*ρ*_^2^ = .27), but no effect of group (*F*(1, 19) < 1, *p* = .52) and crucially, no interaction between noise condition and group (*F*(1, 19) = 1.768, *p* = .20) (Fig. 3a, b) (see also Fig. 3c, d for a summary of individual participants’ slopes). The main effect of threshold was not significant (*F*(1, 19) < 1, *p* = .38), but as in experiment 1, there was a significant interaction between threshold and noise condition (*F*(1, 19) = 11.50, *p* = .003, *η*_*ρ*_^2^ = .38). Again, the relationship between slope and threshold was negative in the comparator noise condition (*r* = −.54, *p* = .009) and positive in the test noise condition (*r* = .50, *p* = .018). Thresholds did not differ significantly between groups (ASC = .25, control = .23) (*t*(20) < 1, *p* = .37).

Finally, a Pearson’s correlation analysis revealed no evidence of a significant relationship between total SRS score and slopes in the comparator or test noise condition (*r < .*16, *p* > .48). In summary, the results of experiment 2 indicate that participants show a bias towards perceiving direct gaze in conditions of uncertainty. However, we found no evidence of any difference in this bias between neurotypical controls and individuals with ASC.

#### Bayesian analysis

A Bayesian independent *t* tests comparing difference scores (noise > comparator) between groups revealed substantial evidence in favour of the null hypothesis (BF_10_ = 3.8) (i.e. no difference between groups).

### General discussion

In two separate experiments, we used a gaze discrimination paradigm to investigate whether priors for direct gaze are diminished in neurotypical individuals with high numbers of autistic traits and in individuals with a clinical diagnosis of high-functioning ASC. In experiment 1, neurotypical individuals showed evidence of a bias towards perceiving gaze as direct under conditions of uncertainty (viewing eyes through noise), consistent with a prior for direct gaze [25]. However, we found no evidence that the strength of the direct gaze bias is reduced in individuals with high numbers of autistic traits. Experiment 2 compared the strength of the direct gaze bias between individuals with ASC and matched neurotypical controls. Both control and ASC groups showed evidence of a direct gaze bias, and the magnitude of this effect did not differ between groups. To our knowledge, this is the first study to empirically test the theory of attenuated priors in ASC [8] in high-level social perceptual processing. Our results support the proposal that humans have a bias towards perceiving gaze as direct [25, 26] but suggest that priors for gaze direction are not diminished in adults with high-functioning ASC.

In both experiments, we found an interaction between threshold and noise condition, reflecting an increase in the magnitude of the direct gaze bias with an increase in stimulus noise. This result accords with a Bayesian framework where increased uncertainty is associated with increased influence of the prior. Furthermore, there was no evidence that sensitivity to noise differed between groups, indicating that an equivalent influence of priors on perception in ASC is unlikely to result from a combination of hypo-priors and increased sensory uncertainty.

One alternative explanation for the findings of this experiment, and previous studies on the direct gaze bias, is that the perceptual shift towards direct gaze reflects a generalised tendency to perceive objects as being centred (in this case, centring the iris in the sclera) rather than a specific prior towards direct gaze. In a previous study, Mareschal et al. [26] addressed this by performing a comparable discrimination task using a grey circle placed within a larger white circle. In contrast with the gaze results, there was only a weak and non-significant bias towards viewing the object as centred (*p* > 0.3). Thus, although some generic process favouring symmetry might have a small role in the direct gaze bias found here, this is unlikely to account for the entire effect.

Brock [43] proposed that a ‘bottom-up’ account of enhanced perception in ASC would lead to similar predictions as the attenuated priors theory. Importantly, this account proposes that priors are not attenuated in ASC. Rather, incoming sensory information is less noisy than in neurotypical individuals (i.e. the variance of the sensory observation is narrower) [43]. With less uncertainty about the noisy sensory observation, the prior has less influence on perception even though its variance is equivalent to the prior in neurotypical individuals. Our current findings do not fit with this proposal either. Moreover, we also estimated sensory noise in gaze detection judgements using an adaptive noise thresholding task. This revealed no evidence of reduced sensory noise in ASC as thresholds were highly similar for the ASC and matched control group in experiment 2, while group thresholds were almost identical for the low and high AQ groups in experiment 1. This finding contrasts with those of a recent study of self-motion perception in ASC [44] that found that adolescents with ASC showed increased sensitivity to visual noise. One possible explanation for this apparent difference is that any increased sensitivity to visual noise in ASC may be less apparent in the context of higher-level stimuli, such as faces, compared to lower-level stimuli such as moving dots.

Zaidel et al. [44] also found evidence for reduced use of priors in adolescents with ASC, a result that accords with the attenuated priors theory of perception in ASC. However, their study specifically investigated the use of recently acquired priors, suggesting that evidence of a difference in the use of prior information between the two studies may be a consequence of differences in the developmental acquisition of priors and/or atypicalities in the rapid acquisition of priors [45]. Similarly, individuals with ASC might possess priors that exert a similar influence to those of neurotypical individuals (as found here) but that develop differently or are applied with less flexibility. One proposal is that autistic atypicalities are a consequence of overly constrained priors, leading to ‘overfitted’ and non-generalizable predictions [46, 47] (and see also Qian and Lipkin’s [48] learning theory style of ASC). Thus, the developmental trajectory of the direct gaze prior could differ for neurotypical individuals and those with ASC (e.g. steeper acquisition in neurotypical individuals) but eventually converge in adulthood. Differences in the development of priors might also explain why diminished adaptation aftereffects for faces in children with ASC [17, 18] are not found in adults with ASC [19–21]. It should also be noted that while differences in the developmental trajectory of priors may explain the absence of an group effect in our study, a recent study, using a non-social task, found that children with ASC show typical adaptation of perceptual causality (i.e. following prolonged exposure to causal events, subsequent perceptual events are more likely to be judged as non-causal) [49]. Thus, any attenuation of priors in children with ASC [17, 18] does not appear to be generalise to both social and non-social domains.

Given the significant heterogeneity of symptoms across individuals with ASC, it is also possible that attenuated priors are only found in a subset of individuals within the autistic spectrum or in specific behavioural domains [50]. In this study, we failed to find any evidence of atypical priors in the domain of social perception, or specifically, perceiving the direction of eye gaze. Whether the development of a prior for direct gaze differs to the development of priors for other types of social information is unclear. For example, a direct gaze prior could be innate or develop in infancy. Baron-Cohen [51] and Mareschal et al. [25] suggest a bias towards direct gaze would offer a social/evolutionary advantage because direct gaze is such an important social cue that often signals an imminent interaction (e.g. attack or pro-social approach). Assuming that gaze is direct when one is unsure would represent a safer strategy than trying to calculate the precise gaze direction [52, 53]. Future work will be needed to determine whether ASC is associated with specific differences in the acquisition and use of newly learned priors and to what extent any differences generalise across both social and non-social domains.

It should be noted that participants with ASC in this study were all high-functioning, with both the ASC and control groups having a high average IQ. It is not known if these findings would generalise to individuals with lower-functioning ASC. In addition, ‘standard’ assessments were not available for all participants included in experiment 2. Despite these caveats, it is important to note that the behavioural atypicalities reported by individuals in our sample (social or otherwise) were sufficient to cause problems in everyday life and lead to significant distress; otherwise, they would not have a clinical diagnosis. This indicates that the presence of typical priors (in at least one domain) does not necessarily signify typical social functioning. Indeed, we found no evidence that the strength of the direct gaze bias is related to the number of self-reported autistic traits on the SRS, a measure of the extent of autistic social impairment [34]. Moreover, despite evidence indicating atypical social perception in ASC, there are a number of studies reporting typical social perception in ASC, including orientating towards facial stimuli and interpreting other people’s actions perception [6, 7]. An alternative reason for the lack of significant group differences in experiment 2 is due to the small sample size used. However, Bayesian analyses revealed substantial evidence in favour of the null hypothesis for experiment 2. Thus, even with a smaller sample size in experiment 2, evidence indicates that both studies show substantial evidence in favour of the null hypothesis, i.e. no difference between groups. Taken together, both experiments indicate that the influence of priors on perception does not differ between groups on the basis of autistic traits or on a clinical diagnosis of high-functioning ASC. However**,** future studies would benefit not only from larger sample sizes but also by including more representative (i.e. lower IQ) and better characterised samples of the autistic population.

Finally, atypical gaze processing is considered a core symptom of ASC, and numerous studies report atypicalities in gaze processing in ASC, such as reduced attention to the eyes [54], differences in following another’s gaze [55] and difficulty in inferring mental states from the eyes [30]. Although any task involving eye gaze contains a strong ‘social’ component, it should be emphasised that the thresholding and gaze priors tasks required participants to make a perceptual discrimination of gaze direction (‘is the second face looking to the left or the right of the first?’), rather than asking them to make an explicit social judgement (e.g. an evaluation of how the gaze relates to them, or inferring the mental states of the face). Thus, intact performance on this task does not imply typical performance on tasks that relate to understanding the communicative intent and emotional significance signalled by direct gaze. Moreover, our results can only speak to the role of perceptual priors in the domain of visual social perception, and further studies will be needed to test the hypo-priors theory using less ‘social’ tasks. It is important to note, however, that Pellicano and Burr [8] make no distinction in the use of perceptual priors between social and non-social stimuli or between different sensory modalities.

## Conclusions

In summary, we found that participants show a bias towards perceiving gaze as direct under conditions of uncertainty, replicating findings by Mareschal et al. [25]. Importantly, however, we found no evidence that the strength of this bias is reduced in neurotypical individuals with high numbers of autistic traits or in individuals with a diagnosis of ASC. These results challenge the attenuated priors theory of ASC [8] and suggest no apparent atypicalities in the use of prior information in gaze perception in adults with high-functioning ASC.

## Abbreviations

AAA: Adult Asperger Assessment
ADOS: Autism Diagnostic Observation Schedule
AQ: Autism Spectrum Quotient
ASC: Autism Spectrum Conditions
SRS: Social Responsiveness Scale
WASI-II: Wechsler Abbreviated Scale of Intelligence – second edition
